# Machine-Readable Database for Genotype-Phenotype Analyses in Rare Diseases Using FKTN-related Congenital Muscular Dystrophies as a Prototype

**DOI:** 10.21203/rs.3.rs-10856264/v1

**Published:** 2026-09-17

**Authors:** Matthew E. Lefkowitz, Sofia G. Eisenberg, Ruili Huang, Uma S. Mudunuri, Robert Leaman, Zhiyong Lu, Svetlana Gorokhova, Sharie J. Haugabook, Elizabeth A. Ottinger, Ann R. Knebel, Donald C. Lo, Carsten G. Bönnemann, A. Reghan Foley

**Affiliations:** National Institutes of Health; National Institutes of Health; National Institutes of Health; National Institutes of Health; National Institutes of Health; National Institutes of Health; Aix Marseille Univ, INSERM, Marseille Medical Genetics, U1251; National Institutes of Health; National Institutes of Health; National Institutes of Health; National Institutes of Health; National Institutes of Health; National Institutes of Health

## Abstract

The *FKTN*-related muscular dystrophies are a subtype of highly heterogeneous, ultra-rare genetic diseases that are part of the α-dystroglycanopathies (αDGs). These disorders present with different prevalences depending on the population and cause a spectrum of severities with frequently severe phenotypes with often congenital presentation (congenital muscular dystrophy). To better understand the impact of specific *FKTN* variants in the affected individuals, we first comprehensively aggregated all publicly known and available data resulting in a database comprising all published literature that details *FKTN* variants and their associated clinical phenotypes. A machine-readable format for the database was achievedvia harmonized data conversion for genotypes and clinical phenotypes. We also developed a clinical severity scale utilizing previously existing metrics as a means of categorizing phenotypic data to facilitate effective genotype/phenotype correlation. This dataset also serves as a pilot for similar data extraction and harmonization in other muscular dystrophies and rare monogenic diseases.

## Introduction

**Figure 1:**
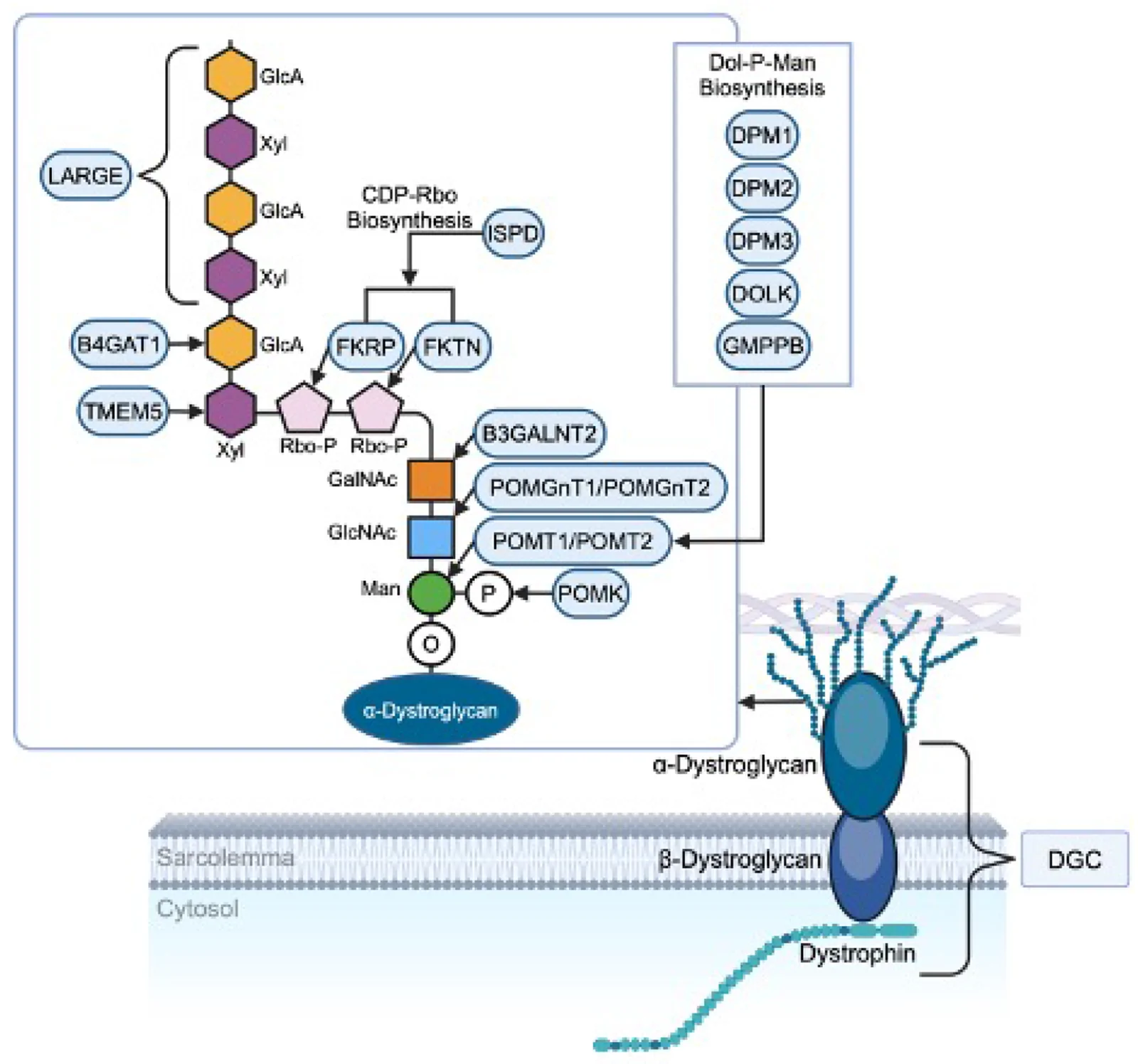
A schematic of the sarcolemma highlighting the dystrophin glycoprotein complex (DGC). Shown in the inset is a glycan chain attached to a-dystroglycan that includes the sugar moieties and the respective genes that are responsible for adding each of the sugars. For the purpose of this work, it is important to focus on the CDP-ribitol biosynthesis pathway where FKTN is responsible for adding a ribitol 5-phosphate group onto the growing sugar chain.

The muscular dystrophies are a diverse group of rare, genetic, neuromuscular disorders which, in many cases, are progressive and result in highly reduced capacities for independent living ^1^. The congenital muscular dystrophies (CMDs) are a category of muscular dystrophies for which symptoms are initially present at birth and often noted during pregnancy; among these are the α-dystroglycanopathies (αDGpathies), disorders which impact the functioning of α-dystroglycan, a key sarcolemmal protein that connects the extracellular matrix with the intracellular actin cytoskeleton ^2–4^. The dystrophin glycoprotein complex (DGC) is a transmembrane protein complex that spans the sarcolemma, with dystrophin binding at the cytosol side, β-dystroglycan spanning the plasma membrane, and α-dystroglycan located on the surface of the cell. Attached to α-dystroglycan are glycan chains composed of specific sugar sequences that interact with ECM proteins, such as laminin. A series of glycosyltransferases and kinases are responsible for adding these sugar moieties to the O-linked specific glycan chain. Pathogenic variants within these genes and resulting proteins lead to defects in α-dystroglycan glycosylation which result in the considerable heterogeneity of the αDGpathies. A specific terminal sugar repeated di-glucoside sequence, termed matriglycan and formed from glycosyltransferases *LARGE1* and *LARGE2*, serve as the end of these chains and act as the receptor with the ECM proteins ^5,6^.The schematic of the glycan chain synthesis is shown in Fig. 1

Among the many genes whose variants can cause a form of αDGpathy, we decided to initially focus on the *fukutin* (*FKTN*) gene to establish our methodology. FKTN is well defined as a biallelic cause of αDGpathy, has a mechanistic understanding to help with variant interpretation, and is rare enough to allow for full capture of the literature. Even though *FKTN* is a gene in which pathogenic variants are still exceedingly rare, there have been nearly 100 studies reported in the literature of αDGpathy related to variants in *FKTN*. The FKTN protein is a ribitol 5-phosphate transferase, which acts in a specific role to glycosylate α-dystroglycan. FKTN’s primary function is to use CDP-ribitol to add a ribitol 5-phosphate (Rbo5P) group to growing glycan chains on α-dystroglycan ^7,8^. Pathogenic variants in the *FKTN* gene can cause a wide array of associated disorders, termed *FKTN*-related CMDs, the most prevalent of which is Fukuyama-type Congenital Muscular Dystrophy (FCMD), exclusively associated with a founder variant in *FKTN.* However, *FKTN*-associated disorders also include phenotypes consistent with muscle-eye-brain disease (MEB), Walker-Warburg Syndrome (WWS), limb-girdle muscular dystrophy (LGMD), and dilated cardiomyopathy (DCM) ^4,9^. Even though FCMD is most typically caused by the Japanese ancestral founder variant, a 3-kb retrotransposon insertion in the 3’ UTR ^10^, over 1,070 sequence variants, including both pathogenic and benign variants (with the majority being benign), are reported in ClinVar ^11^ alone, and an additional 46 are reported in Leiden Open Variation Database (LOVD) ^12^. The vast majority of these variants are protein truncating, with only a handful of missense variants reported to date. Several further founder variants have been described outside of Japan, including among the Ashkenazi Jewish and Turkish populations ^13,14^.

While patients’ clinical presentations can be multisystemic, nearly all patients are affected with muscle weakness of different severity. Other organ systems can be involved, particularly the central nervous system (CNS) and the cardiovascular and ophthalmological systems ^15^. Other disorders from pathogenic variants in *FKTN* present with an array of symptoms that range in severity ^4,9^.

Because the *FKTN*-related CMDs are a set of ultra-rare diseases, knowledge about these nonetheless important conditions is almost exclusively based on case reports and case series, with nearly a hundred manuscripts worldwide that describe numerous and varied clinical phenotypes and molecular features. The reports describe very disparate populations, and the clinical data is largely not harmonized and has not been summarized or cataloged. A comprehensive, harmonized database is needed to better organize this diverse dataset in order to be able to enable predictions about genetic pathogenicity, understand pathogenic mechanisms and clinical pathogenesis, as well as to explore therapeutic opportunities.

To build tools for better pathogenicity correlation and prediction for all *FKTN* variants, here we present our approach to assemble and organize patient data from cases reported in the literature and apply this method to compile a dataset of patients with variations in their *FKTN* gene. Genetic variants were harmonized to a singular format using a multimodal approach with a combination of manual filtering and use of existing bioinformatics tools. A similar process was used to convert qualitative text-based phenotypic information into corresponding identifiers in Human Phenotype Ontology (HPO) format. Finally, we present and apply a new clinical severity scale to patients across our database to generate a comprehensive data element that can bridge the relationship between genotype and phenotype.

## Results

### Manuscript identification totals

**Figure 2:**
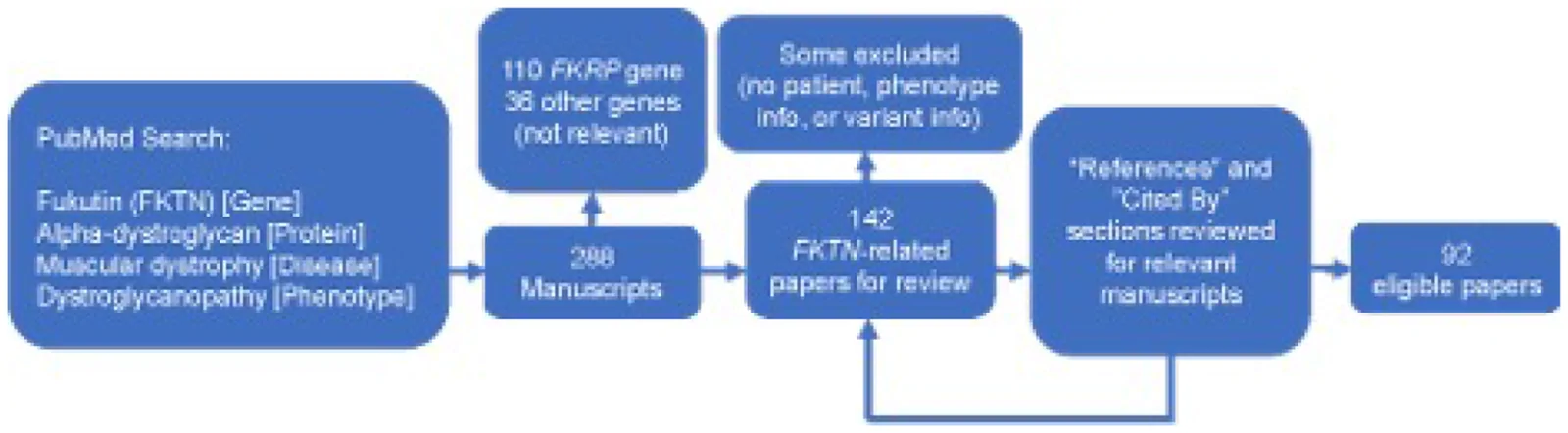
Flowchart showing the manuscript identification procedure. Completing multiple iterations of filtering increased likelihood that all relevant manuscripts were identified.

The search of PubMed identified 288 manuscripts. Of these total manuscripts found, 146 were not relevant to *FKTN*. Of these irrelevant manuscripts, it is interesting to note that 110 were related to the *FKRP* gene, and 36 were related to other genes, many of which are implicated in other secondary dystroglycanopathies. Of the 142 relevant manuscripts, some were excluded because they did not meet the inclusion criteria (detailed above in Materials and Methods). After all search iterations, 92 manuscripts were included, shown in Fig. 2

### Literature Extraction Results

The 92 manuscripts reported on a total of 749 patients, of whom 386 were individual patients from case studies and detailed case series, and 363 patients were from grouped patients from case series. It is important to note that a subset of patients in the catalogue could not be distinguished individually, and they are represented in ranges (“patients in ranges”, or “PIR”). Most of these patients in ranges are from case series.

**Figure 3:**
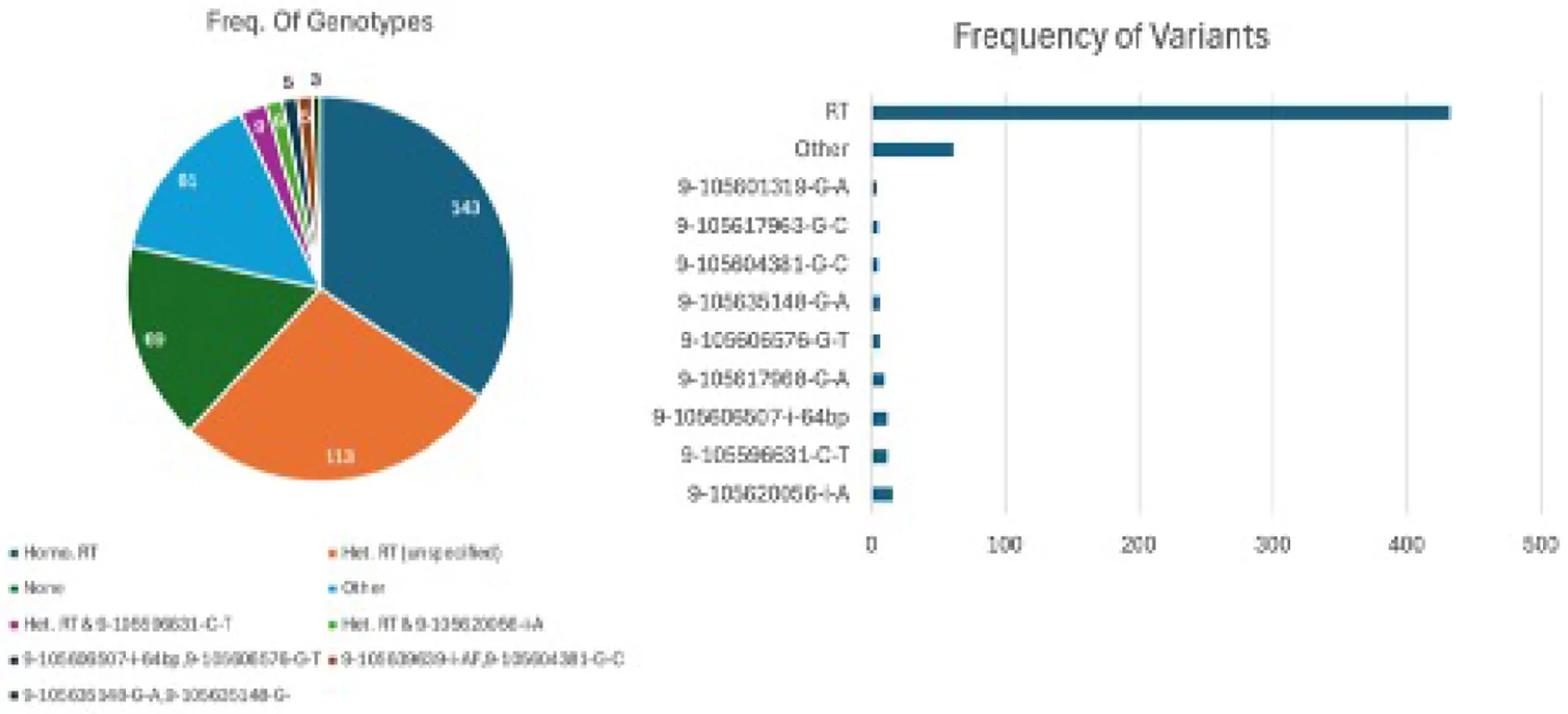
A shows The frequency of genotypes for our patient cohort. Of note, the majority of the cohort is homozygous for the AF genotype. For B, the frequency of unique variants, the retrotransposon (RT) insertion has the highest number of patients.

In total, 52 unique genotype combinations were identified, mostly in a homozygous state. The most common genotype was homozygosity for the Japanese ancestral founder variant, a 3 KB retrotransposon insertion in the 3’ UTR. The Japanese variant is also the most common variant as shown in Fig. 3

As expected, the AF variant was found primarily in patients of Japanese descent, although it was also seen in three Chinese patients and one Korean patient. The Ashkenazi founder variant (NM_001079802.2:c.1167dup) was seen in thirteen patients of Ashkenazi heritage.

Not all manuscripts reported sex, amongst those that did, there was an equal distribution of 109 males and 108 females. If the PIR cases are included, the values were 301 males and 351 females. Sex was not reported in approximately 100 patients. As expected from the large Japanese cohort, the patients were primarily of Japanese ancestry (268). The second-highest ethnicity was Korean, with only 14 patients.

### Variant and Phenotype Harmonization Results

To effectively optimize the dataset for machine readability, we excluded all PIR from the final dataset. After harmonizing the list of variants, we found the number of unique variants matched the original number of unique genotypes from the literature extraction. Ultimately, seven haplotypes were not included because they could not be linked to specific variants. For the phenotypic data, when we employed just NegBio, ~ 60% of non-pertinent positive information was filtered and when the additional scripts were added in conjunction with NegBio, we achieved over 90% of non-pertinent positive information being filtered. In total, the harmonization identified 505 unique HPO terms. After applying the additional filtration of irrelevant terms to the underlying condition, there were 341 total phenotypes.

### Phenotypic Severity

**Figure 4:**
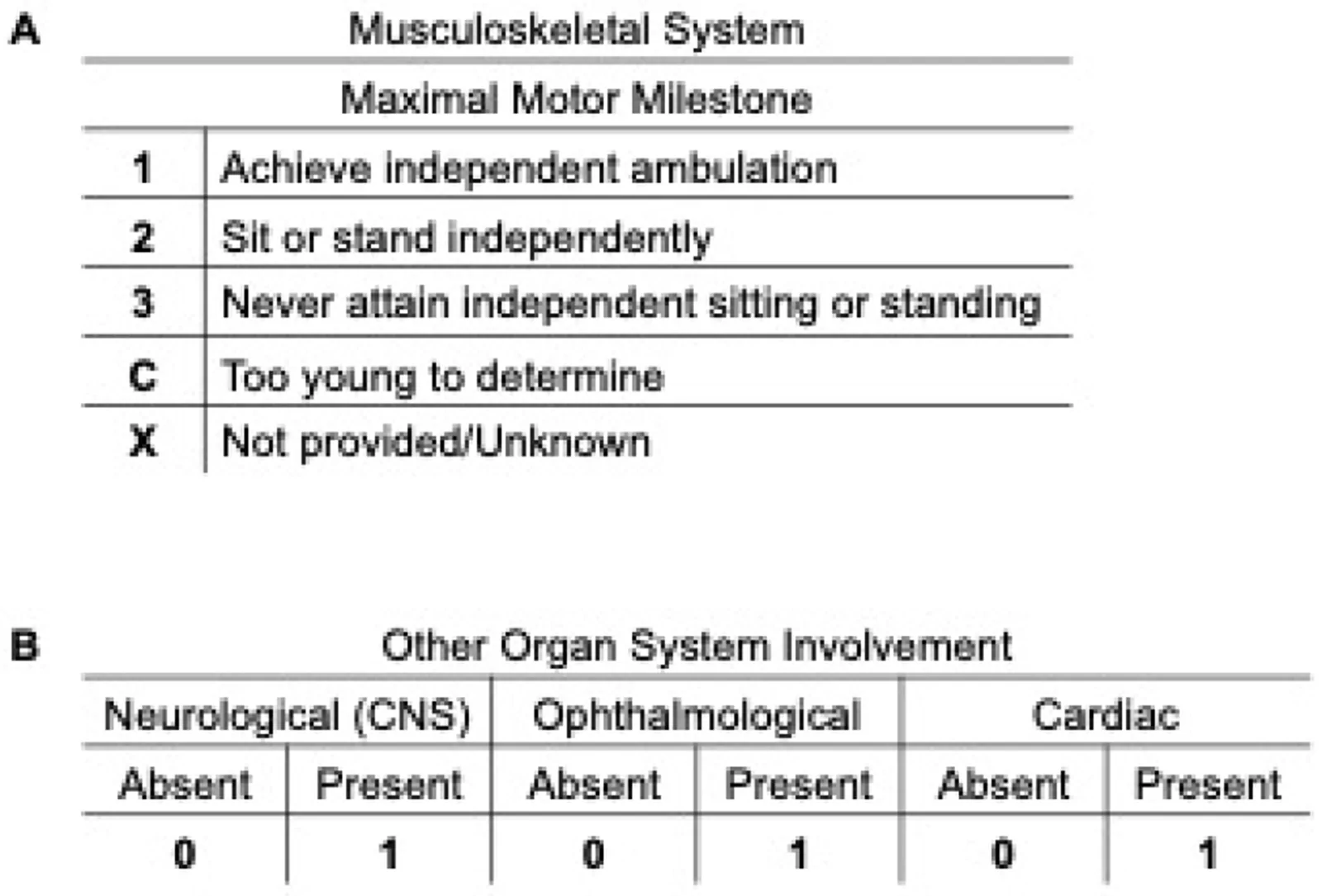
(A) Severity scale based on the maximal motor milestone achieved at one point in the patient’s life, even if the patient loses the ability later. (B) A binary system was used for presence and absence of other organ system involvement including neurological ophthalmological and cardiac systems.

To better characterize and score the phenotypic severity of the patient cohort, a clinical severity scale was created. The scale adapts the modified Ueda classification motor scale ^16^ based on our literature curation and the current understanding of the *FKTN*-related CMDs. Our scale uses a generalized, comprehensive approach based on the maximal motor milestone achieved at some point in the patient’s life, even if the patient subsequently lost that motor ability later in life: “1” represents a mild clinical presentation, describing a patient who achieved independent ambulation; “2” represents a typical, moderate clinical presentation in which a patient achieved the ability to sit or stand independently; and “3” represents a severe clinical presentation, describing a patient who had never attained independent sitting or standing. This category also includes patients that never achieved independent head control. The severity scale is shown in Fig. 4 After developing the clinical severity scale, we then manually assigned each patient a severity level.

For patients with too little information to determine the maximal motor milestone, we developed two distinct categories: “C” represents patients who were too young to determine a maximal motor milestone, including infants for whom this information is lacking or who passed away in infancy; and “X” represents patients for whom there was not enough information to categorize them.

In addition to the motor severity scale, the other main organ systems that are typically involved in *FKTN*-related congenital muscular dystrophies include the neurological (referring to central nervous system or CNS involvement), ophthalmological, and cardiac systems. For these categories, a binary approach was used that indicated presence or absence of clinical pathology organ systems.

**Figure 5:**
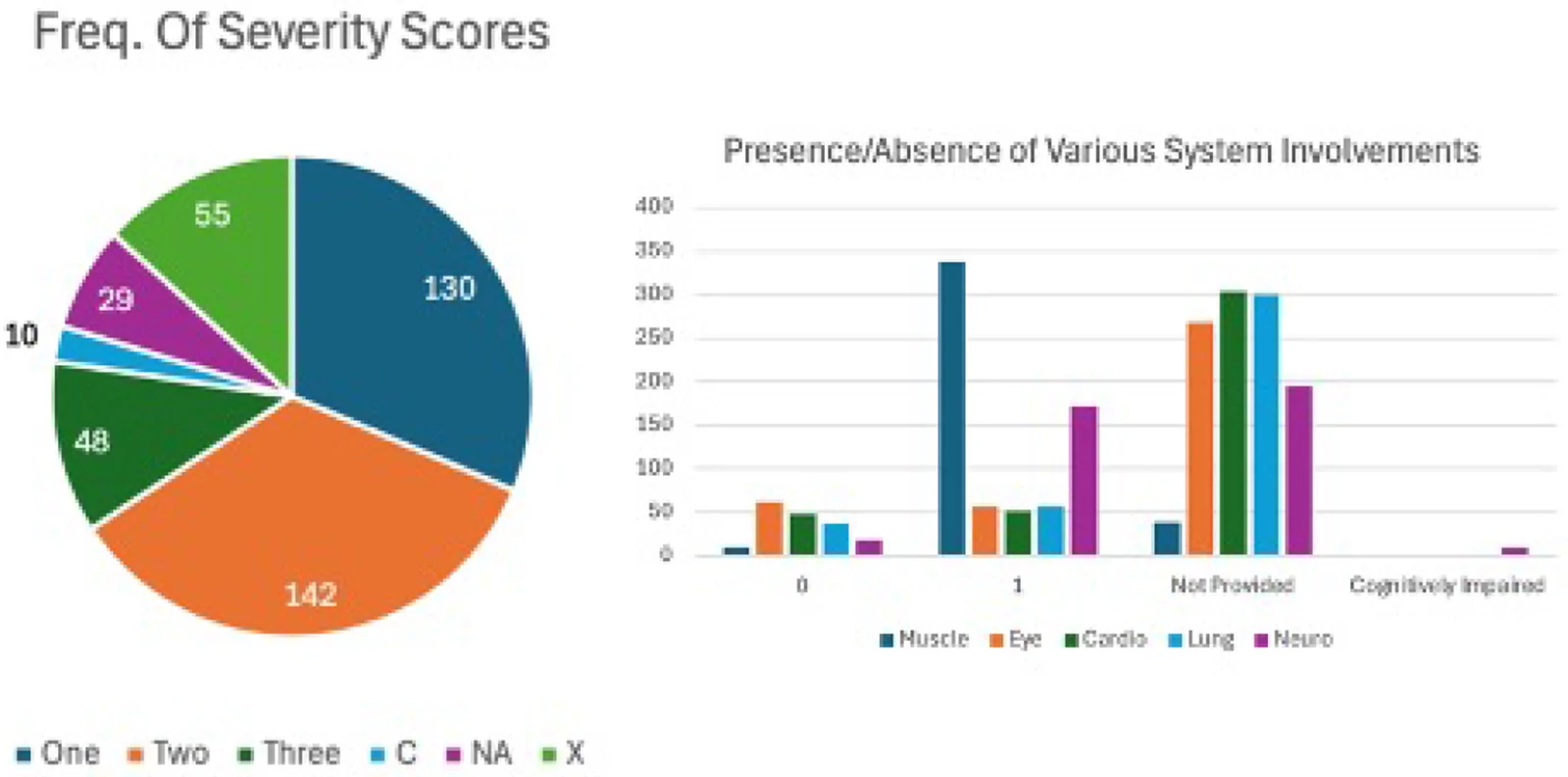
(A) shows the number of patients in the cohort that fall into each severity category. (B) shows the number of patients that have other various organ system involvement.

Using the clinical severity scale described, 60 patients had a mild progression (“1”), 157 had a typical progression (“2”), and 60 had a severe progression (“3”). Of patients without enough information to determine their disease severity, 11 were too young. As expected, the majority of patients (337) were affected by muscle-related symptoms, 173 were affected by neurological symptoms (CNS involvement), 51 by cardiac, and 57 by ophthalmological symptoms. The remainder were either unaffected or did not have information provided. The pictorial representation of the distribution of patient clinical severity is shown in Fig. 5

## Discussion

This work presents a comprehensive literature extraction of *fukutin*-related congenital muscular dystrophy and variants in the *FKTN* gene. We present an exhaustive literature curation and develop a clinical severity scale based on the extracted and aggregated phenotypic information. The data reflect the highly heterogenous nature of *FKTN*-related disorders. While *FKTN*-related CMDs are most typically characterized by a congenital presentation, they can also present later in life. Symptoms range from severe (including death during the neonatal period) to mild, with independently ambulant patients with difficulty climbing stairs. Patients display a range of multi-symptom involvement, including cardiac, neurological (CNS involvement) and ophthalmological symptoms. We especially noticed, across the literature, the very notable impact of febrile illness in aggravating symptoms ^17^.

Moreover, we describe a method for a machine-readable, comprehensive literature extraction and harmonization focused on both genetic and phenotypic patient data. We note that the completeness of our curation of the literature was based on a technique of searching PubMed exclusively, and thus manuscripts which are not available via PubMed would be missed as a limitation of the study. As stated above, we tried to account for this limitation by carefully studying the “Cited By” and “References” sections of all of the manuscripts found via PubMed.

Several techniques were identified as being essential for maintaining the integrity of this data. In particular, when *FKTN* transcript isoform information was not provided (a frequent occurrence), we cross-referenced the variant information with all possible reference sequences. When converting text to HPO terms for machine learning, we employed PhenoTagger in conjunction with either NegBio or similar negation scripts, especially when analyzing natural language. When employing just NegBio, ~ 60% of non-pertinent positive information was filtered and when the additional scripts were added in conjunction with NegBio, we achieved over 90% of non-pertinent positive information being filtered.

These methods utilized in studying the phenotypes and genotypes of *FKTN*-related CMDs can be replicated when working with other ultra rare disorders that have similar numbers of cases reported in the literature (~ 200 papers). There is currently a lack of databases with standardized phenotypic information, especially data with genotype data corresponding to phenotype information. Existing genomic variant databases like LOVD and ClinVar may have limited clinical phenotypic information. There are multiple efforts to develop modern databases and patient registries that use FAIR data principles ^18^. Our data could be easily integrated into these databases to make the old literature data FAIR, thus significantly increasing the number of patients with a rare disease that could be studied/analyzed.

The unique procedures used for the extraction and harmonization of the data for the *FKTN*-related CMDs reported here can be used to analyze the relationships between genotype and phenotype in other rare diseases. Through this work, we were able to develop a clinical severity scale based on the clinical phenotypic data reported from the literature.

When thinking of modern advancements in AI and streamlining complex processes to full automation, such as the ones we employed, we discuss where all the processes we used stand in an effort to achieve autonomous literature extraction and curation. In the data collection stage, we had to manually extract data from the literature into our dataset. We recognize that the path towards automation for this stage will require substantial capability of the AI to be able to: extract individual patient tagging, recognize and extract all variant nomenclatures (including older approaches such as linkage and haplotype analysis), and be able to recognize and extract pertinent phenotypes and symptoms. In the data harmonization and curation stage, we also recognize that the path towards automation will require substantial complexity from the AI to be able to: harmonize outdated variants to the most updated American College of Medical Genetics and Genomics (ACMG) criteria, and to be able to filter and harmonize clinical symptoms and phenotypes to a machine-readable format (such as HPO). To achieve autonomous curation, future studies would need to include building complex large-language models (LLMs) and have them attempt to complete the same procedures we have described and use the output of our semi-automatic approach as a control comparison to train, test, and validate these models. In the meantime, through this work, we hope to establish a “blueprint” that can be used for data curation of all rare diseases that can be utilized by the modern advancements and capabilities of machine-learning and AI.

## Methods

### Manuscript identification

A comprehensive literature search for review of *FKTN* was carried out utilizing an iterative process: PubMed was searched using the terms “fukutin” (FKTN) (gene); “alpha-dystroglycan” (the protein primarily affected); “muscular dystrophy” (disease); “dystroglycanopathy” (phenotype), completing our first search on January 15th, 2022 and our final search was completed on April 16th, 2024. After we identified all of the manuscripts with these terms present, we filtered them using the following inclusion criteria: papers detailing a patient/patients with a variant in their *FKTN* gene as well as clinical presentation data. The rationale for using this criteria was that some (older) manuscripts were published without *FKTN* variant information, with patients having been diagnosed based on their clinical findings.

Subsequent searches were conducted by examining relevant manuscripts’ “References” and “Cited by” sections, using the same inclusion criteria. This process was repeated iteratively until it generated no new papers. We made exceptions to our inclusion criteria for foundational papers of particular historical, clinical, or genetic importance, such as: ^15,19^ and thus their inclusion was deemed to be necessary for a comprehensive literature curation. Our iterative search approach strategy was based on an approach initially described by ^20^ for another rare disease.

### Manual literature extraction

Data was then manually extracted by copying and pasting exactly from the identified manuscripts into a spreadsheet (“catalogue” or “dataset”). The catalogue is ordered by patient, rather than manuscript, such that each row in the dataset corresponds to an individual patient. Patients were assigned identifying numbers (“tags”), preventing duplicate patient entries for patients referenced in multiple manuscripts. The data extracted includes all variants described, as well as all clinical information about biological sex, ethnicity, age of clinical onset (which included initial diagnosis and final diagnosis fields), clinical presentations and manifestations, any treatments tried, and the results of those treatments. We further organized our clinical presentation fields into the four common organ systems associated with the muscular dystrophies: muscle-related; neurological; cardiac; and pulmonary. The phenotypic descriptions were recorded verbatim (i.e., in natural language) to reduce bias.

Data were organized into 42 different categories. We created a set of natural language processing “syntax rules” to aid this process. These rules included: a double slash (//) to separate medical descriptions/events, and an ellipse (…) to signify the deletion of original text. For example, a citation or figure reference. When entering tables, an ellipse (…) separates regular headers, a double colon (::) separates a header from the information in the cell, and a double hyphen (--) separates a main header from a secondary one. Acronyms were explained using parentheses, with the acronym’s key copied from the text verbatim.

We created this very specific set of syntax guidelines to establish a comprehensive set of rules so any future applications of the data, including machine-readable applications such as machine learning, can be taught to understand our data and make it digestible. It is also critical to note that all of the data extracted from the literature was incorporated to our database without any alteration to ensure data integrity and to remain true to the manual extraction. We also chose the specific column headers to accurately and concisely extract all aforementioned, relevant information for each patient. Our catalogue was conceptualized and developed with the assistance from the team that studied *SLC6A8*^20^.

### Variant Harmonization

Our next goal was to achieve machine readability of the dataset. We harmonized all variants to genomic locations in the format Genome Reference Consortium Human Build 38 (GRCh38/HG38). Even though most recent reports used standard Human Genome Variation Society (HGVS) format to report the genetic variants, the older studies used a variety of other formats. A variety of tools were used to standardize the variant data including VariantValidator ^21^, Varsome ^22^, Mutalyzer 3 ^23^, and NCBI Nuccore ^11^. We also used ClinVar ^11^ and LOVD ^12^ as comparative references.

A key issue that arose was that there are 10 distinct annotated *FKTN* transcript isoforms reported in NCBI ^11^, and only rarely were the specific isoforms referred to in the manuscripts. Thus, for all tools, each variant was run against each of the ten available isoform identification numbers for *FKTN*. When conflicts occurred between the tools, preference was given to LOVD and ClinVar, as these databases are clinically validated. If these databases were unable to resolve the conflict, the specifics of each paper were used to attempt to reconstruct the original location. If this failed, preference was given to the location according to the canonical reference variant.

Once a proper location was determined, the variant was converted into standard variant call format (VCF) for SNPs (Chromosome, Start, Reference (Ref) nucleotide value, Alternate (Alt) nucleotide value). For insertions and deletions, this format was modified. An insertion was denoted with an “i” in the “ref allele value” position, and then the inserted nucleotides were placed in the “alt allele value” position. Deletions were marked similarly: with the specific deleted nucleotides in the “ref allele value” position and a “d” in the “alt allele value” position.

Several variants were in haplotype analysis notation (listed as a sequence of numbers corresponding to location and pattern), a system used in earlier genomics research. Other than the haplotype variants that were revisited in ^24^, and the Japanese founder haplotype, these variants could not be harmonized, and were excluded from further analyses.

### Phenotype Harmonization

To populate the phenotypic fields, clinical presentation text from the manuscripts were converted to Human Phenotype Ontology (HPO) terms ^25^ using PhenoTagger ^26^, a natural language processing tool that combines machine learning and dictionary methods to recognize phenotypes mentions in free text ^26^. All fields associated with clinical presentations were consolidated and batch-processed using PhenoTagger and then link them to corresponding HPO terms. The use of HPO provides a comprehensive collection of all human medical manifestations and presentations, each assigned a unique identifier (HPO ID) ^25^.

Initial review of the PhenoTagger output revealed several false-positive associations of phenotypes with patients, primarily arising from the tool’s inability to detect negations. For example, if the original text stated, “X did not show signs of muscle hypotonia”, PhenoTagger incorrectly identified “muscle hypotonia” as a phenotype present. Another common issue involved “negation” over coordinated terms and lists. For example, when the original text stated, “X did not have muscle weakness, microcephaly, or retinal detachment”, PhenoTagger incorrectly labeled all three as being positive phenotypes. To address this issue, we integrated NegBio ^27^, a tool for identifying negated phrases, which was able to employ a NegBio filter to ignore the negated phenotypes. While use of NegBio improved the accuracy of associations of patient phenotypes, some false positive remained. To further refine the data and correct the phenotypes extracted from the output of PhenoTagger and NegBio, we developed a series of scripts^28^ To use these scripts, a dictionary of all possible notations that could be a sign of negation, e.g., “no”, “not”, was created. The script adds each negative notation in the dictionary as either a prefix or postfix to each of the text that triggered the phenotype assignment by PhenoTagger, forming a list of all possible negative terms. It then searches for each of the possible negative terms in the input files and outputs all the text where a negative term is found. Finally, the output from the scripts were double-checked against the PhenoTagger output to see if an HPO ID was assigned to any of the negative terms, which would be considered a false positive. For example, PhenoTagger might have assigned a HPO ID based on the term “able to stand”, but the actual text in the input file was “not able to stand”. The scripts would have flagged this assignment as a false positive by detecting “not able to stand”, which is a combination of a negative notation (i.e., “not”) and a piece of text that triggered a HPO assignment (i.e., “able to stand”), in the input file.

Following the initial extraction, a total of 505 HPO terms were identified. Additional manual review identified several terms, or symptoms, that were not associated with the *FKTN*-related disorders. These were attributed to confounding variables. For example, the term “polyphagia” was identified in some cases but was determined to result from long-term steroid use rather than the underlying *FKTN* mutation. The dataset was then manually curated to remove HPO terms deemed to be unrelated to the primary condition. This quality control assessment was informed by an extensive literature review, ensuring that only phenotypes directly attributable to *FKTN*-related CMDs were retained. The assessment was also performed secondarily by a senior clinician with advanced knowledge in CMDs (ARF).

## Data Availability

The datasets generated and/or analysed during the current study are available at RARe-SOURCE^®^, Integrated Bioinformatics Resource for Rare Diseases RARe-SOURCE^™^, https://raresource.nih.gov The underlying code [and training/validation datasets] for this study is available in Github and can be accessed via this link https://github.com/TX-2017/text-processing.
